# Digital future-self interventions to promote physical activity: perspectives of minimally active middle-aged and older adults

**DOI:** 10.1515/jirspa-2025-0014

**Published:** 2025-10-13

**Authors:** Kristell M. Penfornis, Nienke Nooren, Eline Meijer, Winifred A. Gebhardt, Veronica R. Janssen, Geke D. S. Ludden

**Affiliations:** 4496 Leiden University, Health, Medical & Neuropsychology, Leiden, RA, Netherlands; University of Twente, Enschede, Overste, Netherlands; Leiden University Medical Center, Leiden, ZH, Netherlands; Health, Medical and NeuroPsychology, Leiden University, Leiden, ZH, Netherlands

**Keywords:** future-self intervention, physical activity, mental imagery, avatar, older adults, intervention prototypes

## Abstract

**Objectives:**

Promoting physical activity is crucial for reducing disease risk and improving overall health. This study targeted minimally active individuals aged 45 and older. It aimed to inform the design of effective digital tools for promoting physical activity by exploring their perspectives on two prototype future-self interventions: a mental imagery and an avatar-based approach.

**Methods:**

Three online focus groups (n=10, age range 47–70) were conducted to assess the comprehensibility, acceptability, anticipated effectiveness and preferred formatting of both prototypes.

**Results:**

The prototype for both interventions was deemed comprehensible, but the mental imagery approach was found more appealing and anticipated to be more effective in encouraging physical activity. Participants highlighted the importance of user-friendly, visually engaging, and customizable features in the intervention.

**Conclusions:**

Based on the insights, we recommend prioritizing mental imagery future-self interventions with clear default settings – such as future-self task order – while allowing for personalization to optimize user engagement and effectiveness. Findings from this study provide actionable guidance for developing digital physical activity interventions tailored to minimally active middle-aged to older adults.

## Introduction

Insufficient physical activity (PA) is a leading risk factor for disease and premature death [1], while regular PA enhances physical, cognitive and psychological health [2], [3], [4]. Globally, one-third of individuals are physically inactive [5]. In the Netherlands, only up to 44 % of those above the age of 50 meet PA guidelines [6]. As health risks rise exponentially from age 45 [7], 8], this age group is particularly for PA-promoting interventions. Mobile health (mHealth) offers a promising solution, since it can provide support anytime anywhere [9] and has the capacity to effectively promote PA in older adults [10]. One example is *Perfect Fit*, a Dutch mHealth virtual coaching intervention which aims to promote PA, and was designed with particular attention to individuals aged 45+ [11], 12] .

Health behavior change interventions, like *Perfect Fit*, increasingly incorporate identity-focused components that link healthy behaviors to self-identity. Self-identity, or how individuals perceive themselves, is a significant determinant of PA. Those who view PA as part of their self-identity engage in more frequent, intensive and longer bouts of PA [13], supporting the notion that people tend to behave in ways consistent with their self-identity [14], 15]. Alignment between PA and self-identity can enhance positive expectations for future performance, creating a positive identity-reinforcement loop [16].

Identity-focused interventions often draw on possible self-related theories, which propose that both current and potential future selves can shape behavior [17], 18]. A clear vision of one’s future self provides a framework against which to compare the current self [19], 20]. This activates self-regulatory processes that help individuals to achieve desired future-self states and avoid undesired ones [18], 19], 21], 22]. Thus, future-self interventions show promise in driving behavior change, including increasing PA.

Two common operationalizations of future-self interventions are mental imagery and avatar-based interventions [23]. Mental imagery future-self interventions prompt people to visualize their future selves, typically who they aspire to become (i.e., desired future-self) and/or who they wish to avoid becoming (i.e., undesired future-self). Common tasks include writing about the envisioned future-selves (i.e., verbal future-self tasks) (e.g. [24]) or searching for images which visualize them (i.e., visual future-self tasks) (e.g. [25]). Avatar future-self interventions use graphic technologies to create digital representations of possible future-selves [20]. The working mechanism may involve a so-called ‘Proteus effect’, where individuals adopt behaviors based on their future-self avatars [26]. For example, seeing a slim, fit avatar might encourage increased PA to align with that image. Both mental imagery [24], [27], [28], [29], [30] and avatar future-self interventions [31], [32], [33], [34], [35], [36] have been shown to effectively increase PA – or precursors thereof (e.g., PA-intention or self-efficacy). In addition, mental imagery [37] and avatar-based interventions [33], 36] have been found to be feasible and acceptable by users.

In most studies, future-self interventions were administered to university students [24], 27], [29], [30], [31], [35], 38], 39]. This implies that individuals above 45, who could have much to gain from future-self interventions, have generally been overlooked. Additionally, although digital administration of future-self interventions has recently gained in popularity, there are only a few examples [24], 25], 40], 41] which can guide the development of practical applications such as *Perfect Fit*. Consequently, the aim of the present study is to gather the perspectives of minimally physically active individuals aged 45+ on both types of digital future-self interventions, with the ultimate aim to inform the development of practical applications such as *Perfect Fit*.

## Materials and methods

### Design

In this human centered design study, three online focus groups were conducted with minimally physically active individuals aged 45+ to gather feedback on mental imagery and avatar future-self intervention prototypes. Focus groups were chosen to encourage participants to share experiences and insights in a non-judgmental setting while sparking discussions around their perspectives on the intervention prototypes [42].

### Participants

Participants were recruited through LinkedIn and Facebook, and the personal network of the research team. To be eligible, participants had to be 45 or older, minimally physically active (i.e., not scoring higher than a four on the Brief Physical Activity Assessment Tool [43]) and to speak sufficient Dutch to participate in the focus groups. Although not an inclusion criterion, efforts were made to include both males and females and diverse socioeconomic backgrounds for a better representation of the target group. One person was excluded for exercising daily and two others dropped-out due to problems launching the online meeting and personal reasons respectively. Three online focus groups were held in February 2022 (n=10). Participant pseudonyms and sociodemographic information are summarized in Table 1.

**Table 1:** Pseudonym and sociodemographic characteristics of participants per focus group.

Pseudonym	Gender	Age	BPAAT-score^a^	SEP	Focus group
John	Male	52	2	High	1
Anne	Female	55	1	High	1
Ben	Male	58	2	High	1
Lynda	Female	61	3	Lower	1
Richard	Male	69	3	Middle	2
Alex	Male	47	0	Middle	2
Henry	Male	70	4	High	2
Mary	Female	56	4	Lower	3
James	Male	59	4	Lower	3
Jessica	Female	55	2	Lower	3

PA, physical activity; ^a^BPAAT, Brief Physical Activity Assessment Tool [43], with a potential score ranging from 0 (not physically active at all) to 8 (physically active on most days of the week). A score of four or lower indicates insufficient physical activity.

### Procedure

**Figure 1:**
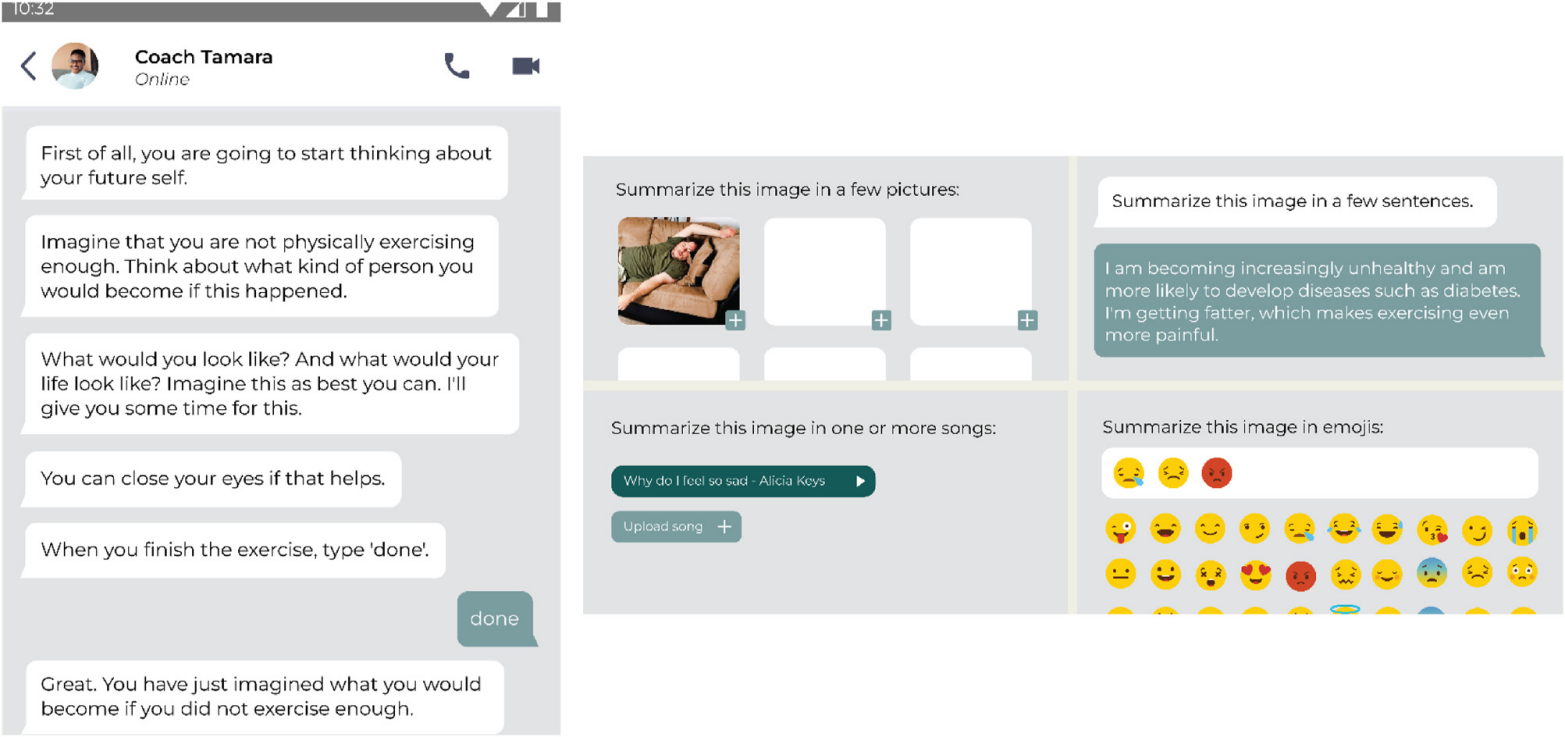
Impression of the mental imagery future-self intervention prototype, featuring the virtual coach’s instructions (left) and four options to describe the envisioned future selves (right).

**Figure 2:**
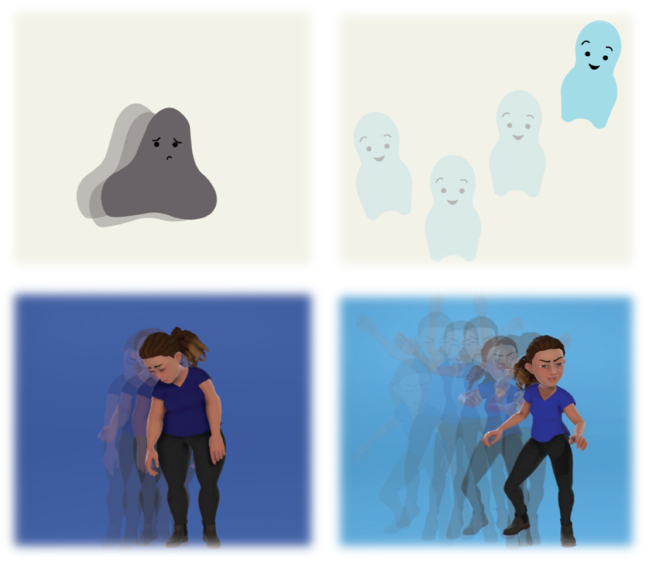
Impression of the video animations shown in the avatar future-self intervention prototype, illustrating possible changes in appearance resulting from increased physical activity for both an abstract (top) and a realistic avatar (bottom).

Before the start of the study, interested individuals received an informational e-mail detailing the study’s overall purpose, voluntary participation and compensation. The e-mail directed them to a Qualtrics questionnaire assessing the eligibility criteria as well as collecting sociodemographic information and digital informed consent. Eligible participants were scheduled for a two-hour focus group based on their availability. Digital prototypes of a mental-imagery future-self intervention and an avatar future-self intervention were developed in Dutch within the present interdisciplinary research team. The team was composed of experts in Human Centered, Interaction and Industrial Design, Health & Medical Psychology, Behavioral Science and the topic of identity in relation to health behaviors. See below for a description, and Figures 1 and 2 for visual impressions of the prototypes. The focus groups were held online via Zoom Video Communications due to COVID-19 restrictions on social gatherings at the time, and led by KP (expertise in Health & Medical Psychology, Behavioral Science and identity research) with NN (expertise in Industrial Design Engineering) as assistant. A semi-structured topic guide and Microsoft PowerPoint presentation were created and can be found in Supplement 1. All three focus groups followed the same structure: introduction of attendees and *Perfect Fit*, information about the focus group’s purpose, presentation and discussion of the two digital future-self intervention prototypes regarding their comprehensibility, acceptability, anticipated effectiveness, and format preferences, and a concluding debriefing. Participants did not use the interventions but were instead asked to share their perspectives on them. Sessions were audio recorded, and attendees were offered a €15 gift voucher for their participation. The study protocol was approved by the Natural Sciences and Engineering Sciences Ethics Committee of the University of Twente (letter number 2021.109). Digital informed consent was obtained from all individuals included in the study.

### Quantitative measures

Sociodemographic characteristics were measured to describe participants in the online questionnaire administered before the focus groups. Year of birth served to determine *age. PA-level* was measured using the Dutch version [44] of the two-item brief physical activity assessment tool (BPAAT) [43], with scores ranging from 0 (not physically active at all) to 8 (physically active on most days of the week). PA score of four or lower indicates insufficient physical activity. Participants were included in the study if they scored four or lower. *Socioeconomic position* was derived from participants’ highest completed education level (as in [45], 46]), recoded as lower (no formal education, primary education and high school or equivalent, lower vocational education), middle (upper secondary education, post-secondary non-tertiary education, tertiary education) or higher SEP (technical/community college, undergraduate, graduate and doctoral degree), based on the Dutch classification of the International Standard Classification of Education (ISCED) [47].

### Qualitative measures

For both prototypes, participants were asked questions about the comprehensibility (e.g., ‘Is the exercise clear?’), acceptability (e.g., ‘What do you think of this exercise?’, ‘Do you have a preference for one exercise?’), anticipated effectiveness (e.g., ‘Would completing this exercise help you to become more physically active?’) and personal format preferences (e.g., ‘How much time would you be willing to spend on creating your avatar?’, ‘Would you rather describe your future-self with words, images, emoji’s or a song?’). Questions about comprehensibility, acceptability, anticipated effectiveness were the same for both intervention prototypes. An overview of all questions asked per prototype can be found in Supplement 1.

### Mental imagery and avatar future-self intervention prototypes

#### Mental imagery future-self intervention prototype

The prototype of the digital mental imagery future-self intervention showed a fictional conversation between the virtual *Perfect Fit* coach and the user. In the conversation, the virtual coach, named Tamara, asked the user to envision their ideal physically active future-self (i.e., desired future-self), and a future-self as insufficiently physically active (i.e., undesired future-self). The content, wording and structure of the desired and undesired tasks was based on prior work conducted within the research group [25], 48] . Then, the user was asked to describe both envisioned future-selves. Four options were shown: verbal description, visualization with (a selection of) images, visualization with emojis, and auditory representation through choosing a song that corresponded to the visualized future-self. See Figure 1 for a visual impression of the conversation between the virtual coach and user and of the four future-self description options.

#### Avatar future-self intervention prototype

Users were asked to imagine that they had logged onto the *Perfect Fit* telephone application and been invited to create an avatar of themselves. They were informed that the avatar would change according to variations in their PA. In the prototype of the avatar future-self intervention, avatar sketches were shown first, ranging from abstract to realistic, varying in appearance and level of detailing. Second, users were shown two video animations of an abstract and a realistic avatar of which the background color, body dimensions, mood and ease of movement changed with increasing or decreasing PA. The content, wording and structure of this intervention was based on an analysis of existing behavior-change interventions [49], [50], [51], [52] and a market analysis of preferred and effective features in existing applications (i.e., Habitica, Smoke Free, Success Life Coach, My Possible Self). The starting appearance of the avatar was the embodiment of a possible undesired future physically inactive future-self while the final appearance of the avatar represented a possible desired physically active future-self. See Figure 2 for a visual impression of the avatar sketches and video animations of the abstract and realistic avatars.

### Data analysis

Data analysis followed the principles of qualitative content analysis [53], using inductive methods. Four themes were defined: comprehensibility, acceptability, anticipated effectiveness and personal format preferences. The analysis was however also data-driven, allowing to identify additional relevant themes.

First, focus group sessions were transcribed verbatim (by NN). Using Atlas. ti version 9, two researchers (NN and KP) independently familiarized themselves with the data and started creating initial codes. An initial thematic framework was developed through discussion between the two researchers and consultation with an additional member of the research team (EM). The framework was then used independently by the two researchers to code the transcripts. One researcher (NN) coded all three transcripts, the other (KP) coded one for reliability purposes. Since most measures were pre-defined questions, coding was quite straight-forward. Agreement between the two coders was high. Disagreements were resolved through discussion between the coders, and discussions allowed to refine the framework. After coding all data, descriptive and numerical accounts of key themes and sub-themes were produced and triangulated to address the research aim. Coding frequencies reported below indicate the number of participants to whom the code(s) applied. As the focus groups followed a semi-structured format, not all participants answered all questions.

## Results

### Perspectives on virtual coaching

During the introduction of the *Perfect Fit* program – before the future-self intervention prototypes were presented – one participant questioned the suitability of an app for individuals aged 45+: ‘*Isn’t this too much from the perspective of young people? While you are trying to guide those over 45? Is it really the case that those over 45 handle a phone with such ease?*’ (Henry). Two participants had a neutral to positive view of app-based virtual coaching, anticipating similar results as with a human coach: ‘*I would have been the one who decided to do this [the* *Perfect Fit* *program]. So then I think I would have no problem accepting artificial intelligence instead of a man/woman. The results will be the same if it [the app] works properly*’ (Ben). Three participants were very negative due to prior negative experiences with, for example, ‘*irritating, unintelligent and unhelpful*’ (James) customer service chatbots. They preferred human contact, believing it essential or preferable to motivate more PA: ‘*I find personal contact more enjoyable than through a robot app*’ (Lynda). There were additional concerns, including the app tracking all movements (n=3) and potentially sending condescending messages if a PA was missed (n=1), reducing enthusiasm about the app. However, these concerns eased after learning that *Perfect Fit* would personalize content, be user-friendly for older adults, and that psychologists were involved in writing positive, motivating messages.

### Perspectives on the mental imagery future-self intervention

#### Comprehensibility

When asked, all participants reported to have understood the purpose of the intervention, and that the instructions given by the *Perfect Fit* virtual coach were clear.

#### Acceptability

The mental imagery future-self intervention appealed to the majority (n=7) of participants, primarily because it made them reflect on a future which they may otherwise not have consciously considered: ‘*You think very quickly about the here and now. How do I feel now? How do I look now? And an exercise that focuses on the future makes you think differently*’ (Mary). Two participants found the intervention unappealing – John felt it was unnecessary, while Henry perceived it as accusatory: ‘*There is something implicitly accusatory in that, something self-accusatory. […] Oh, you see, you’re too fat. That’s your own fault, you should have exercised more and eaten less*.’ Henry’s discomfort stemmed from not identifying with young, fit female coach Tamara. He reported the intervention would feel less condescending if the coach was older and more physically similar to him.

#### Anticipated effectiveness

More than half (n=6) of the participants said that the exercise would stimulate them to increase their PA. Reasons for anticipated effectiveness included that the intervention would prompt reflection about benefits and disadvantages of current and future behavior (n=6), would increase commitment to changing current behavior (n=1), or prompt comparison between the envisioned future-self and the current self (n=1), all of which were anticipated by participants to prompt action. Jessica said that it would be effective because of a sense of accountability towards the coach: ‘*You have a sort of accountability buddy, and that gets you going’*. Some (n=3) expected that the exercise would have no effect on their PA, because they already think about their future regularly and act on this mental image (n=1), or know how to increase their PA (n=2), e.g., ‘*I know what needs to change and I know what I need to do, but for me, it’s just laziness and thinking, ‘tomorrow, the sun will rise again*’ (Lynda). One participant was uncertain about whether it would help increase his PA, but reported to be willing to try it.

#### Format preferences

All participants agreed they would rather complete the intervention within the app, avoiding having to go to external websites. The majority of participants (n=6) preferred receiving all instructions for the intervention at once, allowing them to determine their own reading and completion pace. Regarding the order of future-self tasks, most participants (n=7) would prefer to start with the undesired future-self, in order to end on a positive note or because it would make the desired future-self appear more positive by comparison: ‘*I think that if you first think about the ‘not’, then the ’yes’ comes out more positively, or something like that*’ (Anne). However, two participants would prefer to begin with their desired future-self due to their optimistic nature, while one other would want to focus on just one task – either the desired or the undesired future-self – finding one sufficiently motivating to increase their PA. When it came to describing their future selves, most participants (n=9) favored a visual medium, some (n=4) would opt for a combination of visual and verbal descriptions, one suggested to record a voice memo about the mental image and one, incidentally the youngest participant, preferred using emojis, as they provided a clear depiction of mood. There was no clear preference regarding image selection methods, whether pre-selected, online, or self-produced. Some participants (n=3) expressed that, given their age, they would not like projecting themselves more than one or two years into the future. Additionally, some suggested repeating the mental imagery exercise several times for better results (n=3), receiving reminders of their future selves from the virtual coach (n=2), having audio instructions for the intervention (n=1), or integrating a future-self diary feature in the *Perfect Fit* app (n=1).

### Perspectives on the avatar future-self intervention

#### Comprehensibility

Two participants needed explanation regarding what an avatar is because they were not familiar with the concept: ‘*an ava-what?’* (Henry). Once the term was understood, they did report understanding the intervention and its concept of an avatar which changes with increased or decreased PA.

#### Acceptability

The avatar-related future-self intervention received mixed feedback from participants, despite being described as creative (n=1), humorous (n=2), and visually pleasing (n=1) by some. More than half of the participants (n=6) found it unappealing because it overemphasized body dimensions: ‘*It seems like some kind of weight loss program, as if it is an app for people who weigh 150 kilos and want to reach a healthy weight’* (John). Some (n=3) expressed that a focus on body dimensions made them feel pressured to conform to societal ideals of being slim and fit, which they saw as unattainable and unimportant. Two felt a focus on body dimensions should not be the aim of the *Perfect Fit* app. While neither the large avatar shown at the start of the video prototype nor the fit, muscular avatar shown at the end felt personally relevant to most participants, some (n=2) appreciated that the avatar displayed greater ease of movement, and many (n=7) valued the avatar’s happier appearance at the end: ‘*For me, happier is enough, I think, unless you make it very fat in the first place, then it also helps if it becomes slimmer, but that’s not necessarily required, but rather that it becomes happier*’ (Anne).

#### Anticipated effectiveness

Only two participants, notably the youngest ones, reported that seeing their look-alike avatar change would be motivating to increase their own PA: ‘*If I truly stand behind doing something like that [creating a look-alike avatar], then indeed, I would be more motivated if I could see that my avatar does change*’ (Jessica). Others (n=3) doubted if they would identify with the avatar enough to be motivated: ‘*I don’t really recognize myself in it’* (Richard). One participant felt that alternative methods, such as regular weighing, would be more effective. Furthermore, one participant doubted whether noticeable changes in their avatar could occur within *Perfect Fit*’s limited timeframe: ‘*If I exercise enough for several weeks, I won’t become twice as thin like this*’ (John). Concerns also arose about the potential demotivating effect if the avatar’s progress did not align with real-life results (n=2), or if changes occurred too slowly or too quickly (n=1).

#### Format preferences

Preferences regarding avatar style varied. Half of the participants (n=5) favored a realistic avatar for easier identification, while the other half (n=5) preferred a more abstract version, as it was more appealing and less confronting: ‘*[The abstract one] leaves a lot to your imagination, your own interpretation. The message is very clear, but it’s somewhat less confronting’* (James). Regarding time investment, participants were willing to spend varying amounts of time creating their avatar, ranging from five to ten minutes (n=5), to up to thirty minutes (n=1).

### Comparison of the two future-self interventions

While results indicate a general preference for the mental imagery future-self intervention in terms of appeal and anticipated effectiveness, participants gave mixed responses when asked explicitly. Four participants found the mental imagery exercise more motivating for changing PA-behavior or preferred it over the avatar, which they did not identify with. Five participants favored the avatar exercise for its creative, fun and visual appeal. Of these, three acknowledged that self-monitoring methods without an identity component, like a radial scale with green and red sides or a calendar reminding of PA-goals and tracking activities, could be equally effective in encouraging PA. In other words, only two participants specifically favored the avatar exercise for motivating PA, while to the other three, the specific format of the intervention was less important than its ability to make progress visible. One participant did not favor one over the other, and suggested to administer both, starting with mental imagery and then using avatars to embody the envisioned future-selves.

## Discussion

This study collected the perspectives of minimally active middle-aged to older adults on the comprehensibility, acceptability, anticipated effectiveness, and preferred format of two digital future-self interventions. Findings from this study can be used to inform the effective design and development of digital future-self interventions.

Overall, both the mental imagery and avatar future-self intervention were found to be understandable, appealing and, in varying degrees, anticipated effective in encouraging more PA. This is consistent with possible self-related theory [17], [18], [19] and prior research [23], 24], 27], 31], 33], 36], 37]. It suggests that considering possible future identities is an effective way to guide behavior toward achieving/avoiding desired and undesired identities. However, the mental imagery intervention appealed more, and was anticipated to be more effective in encouraging PA. This may be attributable to adults aged 45+ being less familiar, skilled and/or comfortable with the technology behind avatar-based interventions [54], 55], or needing more time before being comfortable with avatars [56]. The presented avatars may also have felt too normative and focused on body changes, in contrast to mental imagery allowing for greater personal interpretation and tailoring. The use of a young, female avatar may have hindered identification with it, aligning with findings on the Proteus effect [20], 26], 35]. Allowing users to create their own avatars could address this by enabling designs that better reflect their identity and preferences. Alternatively, aligning with findings suggesting that younger participants were more open to using digital technology (e.g. emojis, avatars), avatar future-self interventions may be better suited for younger and middle-aged adults. Finally, avatar future-self interventions may be more effective when matching the personal lifestyle aim(s) of the user (e.g., avatar’s mood improves from increased PA rather its body size changing).

Some participants expressed resistance and distrust towards a digital intervention to promote PA in those aged 45+, for example raising privacy concerns about tracking features and preferring human contact. This aligns with research showing that older adults more often distrust digital health tools and prefer human interactions even when adopting such a tool [57]. However, recent studies found that digital interventions [58] and specifically chatbot coaching interventions [59] can effectively promote PA in older adults. Additionally, some found that older adults are capable of establishing relational bonds with a virtual coaching agent [56]. In light of this, it may be important for digital interventions, such as *Perfect Fit*, to effectively address this resistance in order to maximize uptake and effectiveness of their intervention. To this end, apps using virtual coaches may want to showcase the technology’s intelligent capabilities through, for example, a demonstration video. Studies also suggest giving virtual coaches human-like features (e.g., eyes, mouth) to foster connection [60], though care is needed to avoid misleading users into believing they are interacting with an actual human [61]. Alternatively, incorporating some form of human contact or opting for blended interventions in the future may be viable solutions for this age group. Finally, it may be as simple as giving older users more time to adjust to interactions with virtual agents [56].

Based on our findings, we recommend that digital solutions promoting PA prioritize mental imagery future-self interventions over avatar-based ones. In terms of format, we advise starting with the visualization of the undesired future-self, as seen in comparable interventions such as Functional Imagery Training (see Chapter 3 of [62]), for it allows to end on a positive note. While some preferred having both types of content available, most participants strongly preferred visual over verbal content, such as using images to describe their future-selves or incorporating visual features to make progress visible (e.g., color-coded progress scale). This appeal for including visual content aligns with research indicating that aesthetics significantly enhance app usability and user performance, and improves usability, understanding, enjoyment, uptake and effectiveness of digital health interventions [63], especially in older adults [64]. Thus, we recommend the integration of visual elements in digital future-self interventions. Design guidelines for older users by Gomez-Hernandez et al. [64] may help in this regard. Since, in the present study, preferences varied regarding the order of the future-self tasks and the medium for describing them, users should be allowed to tailor these aspects (e.g., undesired future-self first). Tailoring the projection period into the future is also encouraged. Tailoring has shown to enhance the uptake and effectiveness of behavior change interventions [65] and is expected to benefit digital apps as well. To balance tailoring with development simplicity, intervention developers may consider defining default settings based on the majority’s preference, e.g., desired future-self first, visual descriptions of future-selves using pre-selected images, while allowing individual adjustments.

One key strength of this study is the interdisciplinary collaboration among Health Psychologists, Behavioral Scientists, and Industrial Design professionals. The human-centered design approach also provided valuable insights for tailoring digital future-self interventions promoting PA to minimally physically active middle-aged to older adults. However, our sample was limited to only 10 participants, such that more time may have enabled a larger sample and potentially more robust findings. Nevertheless, prior research shows that 80–90 % of relevant themes can be identified during qualitative analysis with just three focus groups [66]. Second, while falling outside the scope of the present study, we did not explore differences in perspectives across sociodemographic characteristics (e.g., PA-levels, gender, SEP), which could have provided additional design insights regarding what works best for whom. Future studies are encouraged to explore these differences with larger, more diverse samples. For example, the ongoing *Perfect Fit* proof-of-concept study [11], involving 100 participants with varying PA-levels across all SEPs, is expected to shed more light on this particular issue. Lastly, participants only viewed prototypes of the interventions without actively interacting with them, raising questions about the validity of measuring usability, acceptability and anticipated effectiveness (see also [67]). However, given that usability ratings can be stable even after brief exposure to digital content [68] and animated prototypes were used, we believe the impressions formed were sufficient for valid results.

In sum, this study offers valuable insights for designing and implementing digital future-self interventions aiming to promote PA, particularly for minimally physically active middle-aged and older adults. While both mental imagery and avatar-based approaches were clear and understandable, mental imagery stood out as more appealing and likely to encourage increased activity. Findings lay the foundation for further development and refinement of digital health interventions promoting PA. We recommend future-self interventions based on mental imagery, with many visual elements and default settings regarding the order of the future-self tasks, the medium for describing them and projection period into the future. However, it is essential to allow users the flexibility to tailor these features to their preferences. To validate and build on these results, future research with larger, more diverse samples and real-world trials, is encouraged.

## Supplementary Material

Supplementary Material
