# Supplementary material for: Digital future-self interventions to promote physical activity: perspectives of minimally active middle-aged and older adults
Source: J Imag Res Sport Phys Act. 2025 Oct 13;20(1):20250014. doi: 10.1515/jirspa-2025-0014 (PMC12637370; doi:10.1515/jirspa-2025-0014)
Supplement: Supplementary file 1 — Supplementary Material [file j_jirspa-2025-0014_suppl_001.docx]

Supplement 1 – Questions asked during the focus groups regarding the mental imagery and avatar future-self intervention prototypes

| <!--Col Count:3-->Domain of interest | Mental imagery future-self intervention | Avatar future-self intervention |
| --- | --- | --- |
| Comprehensibility | - Is it clear what the exercise entails? If not, can you elaborate on what was is not clear?  - Are the instructions clear? | - Is it clear what the exercise entails? If not, can you elaborate on what was is not clear? |
| Acceptability of the intervention | - What did you think of this exercise? | - What did you think of this exercise?  - How would you feel if your avatar did not change with you? |
| Expected effectiveness in leading to increased PA | - Do you think this exercise would help you to be more physically active?<!--Soft-enter replaced as Paramark-->  - If so, which part do you think would help you the most? | - Would you be motivated to increase your own physical activity if you saw your avatar become happier/more muscular/slimmer and moving more easily by being more physically active? |
| Format preferences | - Would you prefer to first think about who/how you want to be in the future, or who/how you do not want to be in the future?  - Suppose you have just imagined who you have become if you move less in the future, and you are asked to describe that image. Would you prefer to describe it with pictures, write about it, find a song for it, or use emojis for it? Why? Or would you prefer to describe it in another way (besides these 4 options)?  - What would you prefer: 1) instructions that appear sentence by sentence, 2) instructions that appear in pairs or groups of three sentences, or 3) all instructions at once? Why?  - What would you think if you had to do this exercise via an external link that takes you out of the app to a website?  - Can the exercise be improved? If so, how? | - What did you think of the changing abstract and realistic avatars in the videos?  - On the scale from abstract to realistic, which avatar appeals to you the most? Why?  - How would you feel about creating an avatar when you first log in?  - How much time would you be willing to spend creating an avatar?  - Can the exercise be improved? If so, how? |
| Comparison of the future-self interventions | - Which exercise do you prefer? Thinking about yourself in the future or creating an avatar and seeing it change alongside your physical activity?  - Which exercise would help you the most to be more active? | |
